# The role of B3GNT3 as an oncogene in the growth, invasion and migration of esophageal cancer cells

**DOI:** 10.1590/acb380923

**Published:** 2023-03-24

**Authors:** Jiaju Lu, Ting Lei, Haichuan Yu, Xiaojie Su, Lu Zhang, Yu Zhang

**Affiliations:** 1 Lanzhou University – The First School of Clinical Medicine – The First Hospital – Lanzhou (Gansu), China.

**Keywords:** Esophageal Squamous Cell Carcinoma, Transcriptome, Cell Proliferation, Cell Migration Assays

## Abstract

**Purpose:**

To investigate the role and mechanism of β1,3-N-acetylglucosaminyltransferase-3 gene (B3GNT3) in esophageal cancer (ESCA).

**Methods:**

The starBase database was used to evaluate the expression of B3GNT3. B3GNT3 function was measured using KYSE-30 and KYSE-410 cells of esophageal squamous cell carcinoma (ESCC) cell lines. The mRNA levels were detected by quantitative real-time polymerase chain reaction (qRT-PCR). Cell counting kit-8, clone formation assay and transwell assay were used to detect the changes of proliferation, invasion and migration.

**Results:**

B3GNT3 expression was higher in ESCA tissues than in normal tissues. The overall survival rate of ESCA patients with high B3GNT3 expression was lower than that of ESCA patients with low B3GNT3 expression. In vitro functional experiments showed that the proliferation ability, migration and invasion ability of KYSE-30 and KYSE-410 cells with B3GNT3 interference were lower than those of the control, and the overexpression of B3GNT3 had the opposite effect. After silencing B3GNT3 expression in ESCC cell lines, the growth of both cell lines was inhibited and the invasiveness was decreased. Knockdown of B3GNT3 reduced the growth rate and Ki-67 expression level.

**Conclusions:**

B3GNT3, as an oncogene, may promote the growth, invasion and migration of ESCC cell.

## Introduction

Esophageal cancer (ESCA) is a malignant tumor of the digestive system, which is divided into two pathological types: esophageal squamous cell carcinoma (ESCC) and esophageal adenocarcinoma (EAC). Among them, ESCC accounts for the largest proportion, and has the characteristics of strong invasion, rapid disease progression, high recurrence rate and low survival rate^1^^–^ 5. In recent years, the incidence and detection rate of ESCA have increased year by year^6^^,^ 7. The poor prognosis of ESCA is mainly due to its high metastasis rate. Therefore, it is important to determine the molecular mechanisms of ESCA proliferation and metastasis for the development of new therapeutic strategies for ESCA.

The β1,3-N-acetylglucosaminyltransferase-3 gene (B3GNT3) is a member of β-1, 3-N-acetylglucosamine transferase family^8^^–^ 12, a type I transmembrane protein that has been shown to induce apoptosis, cell cycle arrest, autophagy, inhibition of angiogenesis^13^^–^ 16. B3GNT3 is basically expressed in a variety of normal tissues, including stomach, small intestine, colon and placenta^17^^–^ 19. Previous studies have shown that B3GNT3 is highly expressed in a variety of malignant tumors such as nonsmall cell lung cancer, cervical cancer, endometrial cancer, and breast cancer^13^^–^ 15 ^,^ 20. Zhang W et al. reported that B3GNT3 was associated with pelvic lymph node metastasis and poor prognosis in cervical cancer^15^. Studies have also shown that silencing the expression of B3GNT3 can inhibit the proliferation and invasion of lung cancer cells^21^. Barkeer et al. have confirmed that B3GNT3 is highly expressed in pancreatic cancer stem cells and can up-regulate the expression of pancreatic cancer stem cell-related markers, thereby maintaining its stemness^22^. Wang J et al. indicated that B3GNT3 may promote the growth, invasion and migration of endometrial cancer cells by regulating markers related to the RhoA/RAC1 signaling pathway, suggesting that B3GNT3 may be a candidate biomarker for EC therapeutic intervention^23^. At present, no study has systematically reported the expression pattern and clinical significance of B3GNT3 in ESCA. Therefore, exploring the role of B3GNT3 in the progression and tumorigenesis of ESCA may help to further understand the regulatory mechanism of the potential signaling pathway of ESCA tumorigenesis, and provide new ideas and more efficient targets for blocking ESCA.

In view of these, from the molecular level in this paper, the mechanism of occurrence, development and metastasis of ESCA, this study reveals the biological relationship between cell type and cell specificity in ESCA tissues targeted by B3GNT3, which will provide more reference for the development of potential anti-ESCA drugs in the future.

## Methods

### Bioinformatics analysis

From starBase database (https://starbase.sysu.edu.cn/panCancer.php) to download the clinical information of all samples (including age, sex, overall survival, tumor and installment) and RNA-Seq data, a total of 126 cases of ESCA. In addition, the datasets were externally validated.

### Cell lines and cell culture

Human ESCC cell lines KYSE-30, KYSE-410 were procured from Procell (Wuhan, China) and cultured in Roswell Park Memorial Institute-1640 (RPMI-1640) (Procell, Wuhan, China) medium, supplemented with 10% fetal bovine serum (P20522, TRAN, China) and 1% penicillin-streptomycin under the atmosphere of 5% CO_2_ at 37 °C. The human normal esophageal epithelial cells cultured in the atmosphere 5% CO_2_ at 37 °C (HEEC, WheLab, Shanghai, China) were regarded as control to detect the mRNA expression of B3GNT3.

### Quantitative real-time polymerase chain reaction (PCR)

Total RNA was isolated from cultured cells with RNA Trizol Reagent (Hefei bomei, Anhui, China). Reverse transcription was performed by a PrimeScript RT reagent Kit (TaKaRa). Beta-actin as an internal control, was adopted to normalize the mRNA level of B3GNT3. The cycle threshold (CT) values of each test sample during the PCR process were analyzed using Thermo Scientific PikoReal software. The relative mRNA expression level was calculated by 2^-∆∆CT^. Primer sequences are shown below:

B3GNT3

F: 5’- CTCTTCAGTCTGCTAGTGTCAC -3’;

R: 5’- TGTACAGGAGGAAGTTCTGAAC -3’.

GAPDH

F: 5’- TGACTTCAACAGCGACACCCA -3’;

R: 5’- CACCCTGTTGCTGTAGCCAAA -3’.

### Western blot (WB)

Protein of cells was extracted by radio immunoprecipitation assay lysis buffer. A bicinchoninic acid protein assay kit (Beyotime, Jiangsu, China) was used to determine proteins’ concentration. Equal quantity of proteins (20 μg) was separated with 10% sodium dodecyl sulfate-polyacrylamide gel electrophoresis and then transferred onto polyvinylidene fluoride membranes. Upon blocking with 5% skimmed milk powder for 1 h, the blots were probed with the appropriated primary antibodies anti-B3GNT3 (A17611, abclonal, Wuhan, China) at 4 °C for the night. Beta-actin was regarded as an internal reference. Subsequently, these samples were incubated with the corresponding secondary antibodies (biotinylated goat anti-immune IgG, ab6721, Abcam, Shanghai, China) for 1 h at room temperature. The polyvinylidene fluoride membrane was tiled onto the exposure plate, and the electrochemiluminescence luminescent solution was dropped and reacted for 1 min before exposure. The bands were scanned by exposure scanning with Tianeng GIS chassis control software V2.0, and the results were expressed as the relative expression of target proteins.

### Cell proliferation assay

Cell proliferation was evaluated by cell counting kit-8 (CCK-8) and clone formation experiments. KYSE-30 and KYSE-410 cells in logarithmic growth phase were seeded into 96-well plates at a density of 1 × 10^4^ cells per well after transfection for 24 h. After the cells adhered to the wall, the groups were set as follows: Experiment 1: si-NC group, si-B3GNT3#1 group, si-B3GNT3#2 group, si-B3GNT3#3 group, 10 μL CCK-8 reagent (Beyotime, JiangSu, China) was supplied to the cell culture medium and cultured at 37 °C with 5% CO_2_ for 24 h. Experiment 2: ov-NC group and ov-B3GNT3 group, the corresponding amount of virus was added according to the multiplicity of infection and virus titer of cells, and the cells were infected according to the grouping, and cultured at 37 °C and 5% CO_2_ at a constant temperature. Sixteen hours after infection, the cultures were exchanged with complete medium and incubated at 37 °C with 5% CO_2_ for 72 h. Then, the optical density value was detected by a microplate reader at 450 nm.

As for clone formation assay, after transfection, cells were seeded into 35 mm dishes and incubated in complete medium at 37 °C with 5% CO_2_. After continuous culture for 14 days, the cells were washed with phosphate buffered saline twice and fixed with 4% paraformaldehyde for 30 min, following by staining with 0.1% crystal violet solution for 30 min. After air-dried, the colonies were photographed and counted. si-NC were purchased from Thermo Fisher Scientific (4390843, Thermo, Shanghai, China).

The siRNAs used were:

si-B3GNT3#1

F: 5’-CAGACAACAUGGUCUUCUACC -3’;

R: 5’- UAGAAGACCAUGUUGUCUGUG -3’.

si-B3GNT3#2

F: 5’-CCUCCUCUUCAGUCUGCUAGU -3’;

R: 5’-UAGCAGACUGAAGAGGAGGAG -3’.

si-B3GNT3#3

5’-AGCAGGUCCUGUUCUUACAGUT -3’;

UGUAAGAACAGGACCUGCUUG -3’.

### Transwell invasion assays

Transwell assay was used for cell invasion assay. Cells (2 × 10^5^) were suspended in serum-free medium and plated in triplicate in 6-well plates precoated with Matrigel and incubated for 4 h; 100 μL of prewarmed serum-free medium was added to the upper chamber and allowed to stand for 20 min at room temperature until the remaining culture medium was removed by suction. The transfected KYSE-30 and KYSE-410 cells that were starved before the experiment were taken, and 600 μL complete medium containing 20% FBS was added to the 24-well plate lower chamber; 200 μL of cell suspension was added to the transwell chamber and placed in the incubator for 24 h. Then, the cell compartment was fixed with ethanol and stained with 0.5% crystal violet for 30 min. Images of the invading cells were obtained by an inverted microscope (DMI1, LEICA, Germany). Three visual fields were randomly observed in each group.

### Cell migration assay

To estimate the migrative ability of cells, 2 × 10^5^ cells were seeded into a 6-well plate for wound-healing assay. Twenty-fourhours later, the wound width was determined using an inverted microscope (DMI1, LEICA, Germany). To estimate the migrative ability of cells, upon incubation for 24 h, cells on the lower surface were washed with phosphate buffered saline, fixed with 4% paraformaldehyde and stained with 0.1 % crystal violet solution. Finally, three fields of view were randomly selected and cells were photographed and counted under a light microscope.

### Flow cytometry

Cell apoptosis was measured by Flow cytometry. Cells were harvested by trypsinization without EDTA and seeded into a 6-well plate at a density of 5.0 × 10^4^ cells; 5 μL of annexin V and 5 μL propidium iodide were added to each cell suspension, and incubated at room temperature in the dark for 15 min. Then the samples were analyzed by flow cytometry (Cytoflex, Beckman, China).

### The subcutaneous xenograft tumor model of ESCA in mice was prepared

KYSE-30 cells in logarithmic growth phase were digested with trypsin and transferred to a 5 mL centrifuge tube. The cells were separated at 100 rpm for 10 min at 4 °C, and the supernatant was discarded. Cells were then resuspended in serum-free medium. The cell suspension was adjusted to a concentration of 1 × 10^7^ mL^–1^, and the cell suspension was thoroughly mixed with the BD Matrigel matrix gel in a 1:1 ratio. The ESCA subcutaneous transplantation tumor model was established by injecting 0.2 mL of the KYSE-30 cell suspension prepared above into the right armpit of the BALB/c nude mice forelimb. The other mice were injected with normal saline according to the same procedure and were set as the blank group. Five successfully transplanted mice were cultured under specific pathogen-free conditions, and all experiments were approved by the Ethics Committee of the First Hospital of Lanzhou University and performed in accordance with institutional guidance, Ethics No: LDYYLL2022-355. The body weight of mice was recorded every week after inoculation, and the long diameter (L) and short diameter (W) of mice tumors were measured using vernier calipers, and the tumor volume was calculated as (L × W^2^ / 2). Plasmid negative control (NC) and lentiviral short hairpin RNA targeting B3GNT3 (sh-B3GNT3) were purchased from Hewu Biotechnology Co., Ltd. (P15297, Hewu, Shanghai, China).

### Anatomical and pathological observation

The mice with a survival time of more than 30 days were killed by euthanasia (potassium chloride injection), and the axillary tumors were quickly isolated, and the blood stains were washed. The formation of metastatic lesions was observed and recorded, and the expression of B3GNT3 was detected by qRT-PCR and WB. At the same time, 5 mice were used as negative control.

### Immunohistochemical staining was performed

Paraffin sections were prepared, dewaxed, hydrated, antigen repaired, endogenous peroxidase blocked, Ki-67 antibody reagent was added, goat serum was added, chromogenic agent was added, counter-stained, dehydrated, transparent and sealed. After the Ki-67 immunohistochemical staining was completed, images were collected with a high-power imaging microscope of image acquisition software, and the acquired images were analyzed.

### Transcriptome sequencing

RNA extracted by Trizol method was sequenced by Illumina HiSeq sequencing system, and the quality of RNA samples was evaluated. The mRNA with poly A tail was enriched by magnetic beads. rRNA was hybridized with a DNA probe, RNase H was used to digest the DNA/RNA hybrid strand, and then DNAse I was used to digest the DNA probe. After purification, the required RNA was obtained, connected the 3’ end and 5’ end of the linker successively, reverse transcribed into cDNA, and amplified by PCR. Then the target fragment library was cut and recovered using the gel recovery kit, and the library with qualified quality inspection was sequenced.

### Bioinformatics analysis

The raw data obtained by sequencing were filtered first: the joints at both ends of reads and the reads with fragment length < 17 nucleotides and low quality were removed to complete the preliminary filtering of data and obtain high-quality data (clean reads). The distribution map of genome-wide reads was obtained by comparing clean reads with the reference genome, and the clean reads were annotated by noncoding RNA classification. The expression of identified mRNA was calculated and differentially expressed mRNA among samples were analyzed. Target genes of mRNA with significant differences were further predicted, and l (GO, http://geneontology.org/) and Kyoto Encyclopedia of Genes and Genomes (KEGG, http://www.kegg.jp/) biological pathway enrichment analysis were performed on target genes.

### Statistical analysis

Statistical analyses were performed using GraphPad Prism 8.3.0 software and SPSS 26 software. The Student’s t-test was used for the comparison between two groups; one-way analysis of variance was used for comparison with more than two groups. The p < 0.05 was considered as statistically significant for all tests.

## Results

### The effect of B3GNT3 expression on ESCA cell

**Figure 1:**
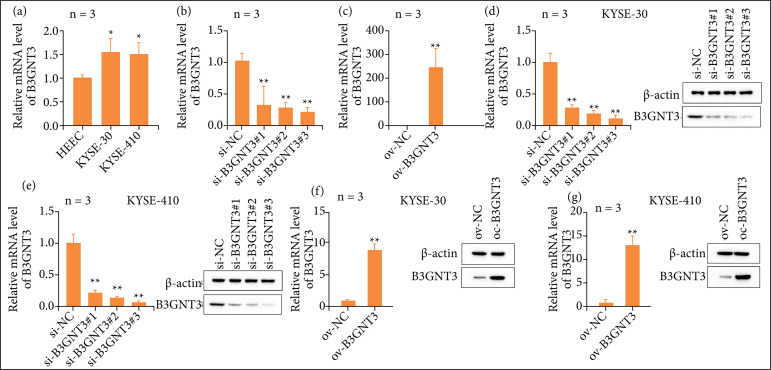
Expression of B3GNT3 in ESCA. **(a)** QRT-PCR was used to determine the mRNA expression of B3GNT3 in three ESCA cell lines. * p < 0.05 vs. HEEC. **(b)** QRT-PCR was used to determine the mRNA expression of si-B3GNT3 in three ESCA cell lines. ** p < 0.01 vs. si-NC. **(c)** QRT-PCR was used to determine the mRNA expression of ov-B3GNT3. ** p < 0.01 vs. ov-NC. **(d)** WB was used to determine the mRNA expression of si-B3GNT3 in KYSE-30 cell lines. ** p < 0.01 vs. si-NC. **(e)** WB was used to determine the mRNA expression of si-B3GNT3 in KYSE-410 cell lines. ** p < 0.01 vs. si-NC. **(f)** WB was used to determine the mRNA expression of B3GNT3 in KYSE-30 cell lines. ** p < 0.01 vs. ov-NC. **(g)** WB was used to determine the mRNA expression of B3GNT3 in KYSE-410 cell lines. ** p < 0.01 vs. ov-NC.

The expression of B3GNT3 in ESCA cells was detected by QRT-PCR, and compared with normal cells, it was found that the expression of B3GNT3 in two ESCC cell lines were higher than that in normal HEEC cell line (p < 0.05, Fig. 1a). To further investigate the significance of B3GNT3 expression in ESCA, B3GNT3 small RNA silencing and overexpression adenovirus were constructed. The transfection efficiency of B3GNT3 siRNA was first detected by qRT-PCR and further observed by WB that B3GNT3 mRNA levels were significantly reduced in the si-B3GNT3 group compared with the si-NC group in HEEC, KYSE-30 and KYSE-410 cells (p < 0.01, Fig. 1b, d, e). Besides, the protein levels of B3GNT3 were also increased after overexpression of B3GNT3 compared with ov-NC groups (p < 0.01, Fig. 1c, f, g). The above results indicate that B3GNT3 may be a key oncogene in the occurrence and development of ESCA.

### The effect of B3GNT3 expression on ESCC cell proliferation

**Figure 2:**
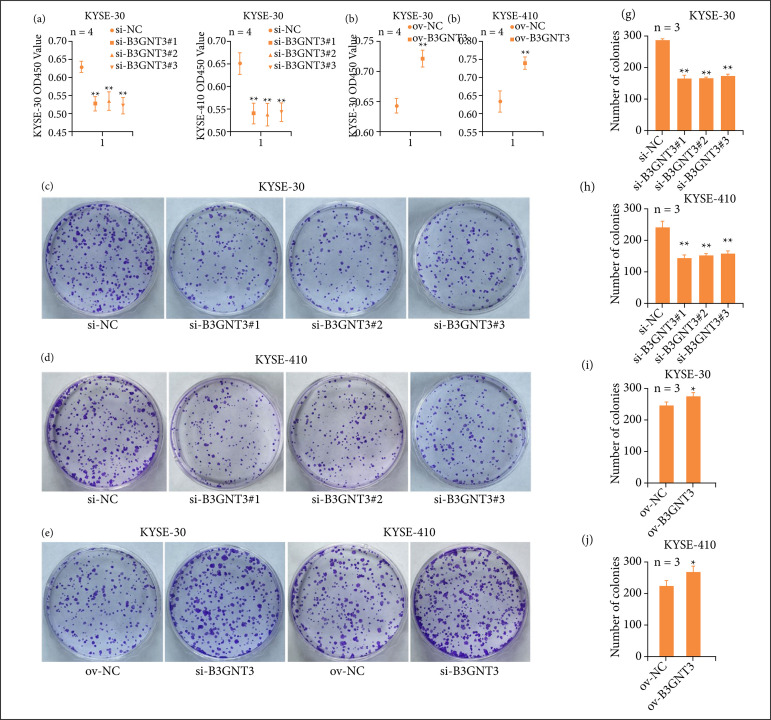
Effects of B3GNT3 on ESCC cell viability. CCK-8 **(a, b)** and clone formation **(c-j)** assays were used to detect the proliferative capacities of KYSE-30 **(c, e, g, i)** and KYSE-410 **(d, f, h, j)** cells. **p < 0.01 vs. si-NC group, *p < 0.05 vs ov-NC group.

To explore the biological function of B3GNT3 in ESCA, the effect of B3GNT3 on ESCC cells were first evaluated by CCK-8 and clone formation experiments. According to the CCK-8 assays, compared with si-NC group, the optical density values in KYSE-30 and KYSE-410 cells was notably reduced when B3GNT3 was downregulated (p < 0.01, Fig. 2a). Meanwhile, the optical density values were significantly elevated after overexpression B3GNT3 (p < 0.01, Fig. 2b). In agreement with the above results, compared with si-NC group, the clone formation capacity of KYSE-30 and KYSE-410 cells were significantly reduced (p < 0.01, Fig. 2c, d, g, h). After overexpression B3GNT3, the number of colonies were increased. (p < 0.05, Fig. 2e, f, i, j). All these data suggested that B3GNT3 could promote the proliferation of ESCC cells.

### The effect of B3GNT3 expression on ESCC cell migration and invasion

**Figure 3:**
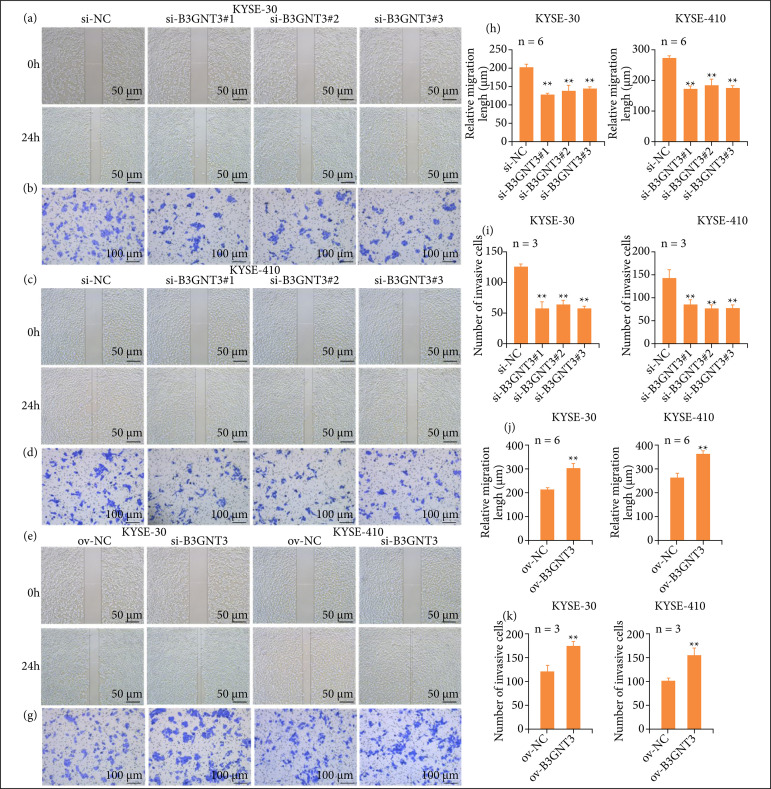
B3GNT3 influences migration and invasion of ESCC cells. Migration ability of KYSE-30 and KYSE-410 cells was estimated using a wound healing assay **(a, c, e, f, h, j)**. Scale bar, 50 μm; ** p < 0.01 vs. si-NC. Cell invasion was determined by transwell assay **(b, d, g, i, k)**. Scale bar, 100 μm; ** p < 0.01 vs. ov-NC.

The effect of B3GNT3 on the migration and invasion of ESCC cell lines was detected by the would-healing and transwell assay. The would-healing assay revealed that cell migration was restrained in KYSE-30 and KYSE-410 cells (p < 0.01, Fig. 3a, c, h). Consistently, overexpression of B3GNT3 promoted cell migration, supported by the significantly increase migration cell number in (p < 0.01, Fig. 3e, f, j). Similarly, the invasiveness of KYSE-30 and KYSE-410 cells also was decrease (p < 0.01, Fig. 3b, d, i). Furthermore, overexpression of B3GNT3 in KYSE-30 and KYSE-410 cells enhanced the cell migration and invasion in the transwell assay (p < 0.01, Fig. 3g, k). Taken together, our findings indicated that B3GNT3 influences migration, and invasion of ESCA cells.

### The effect of B3GNT3 expression on ESCA cell apoptosis

**Figure 4:**
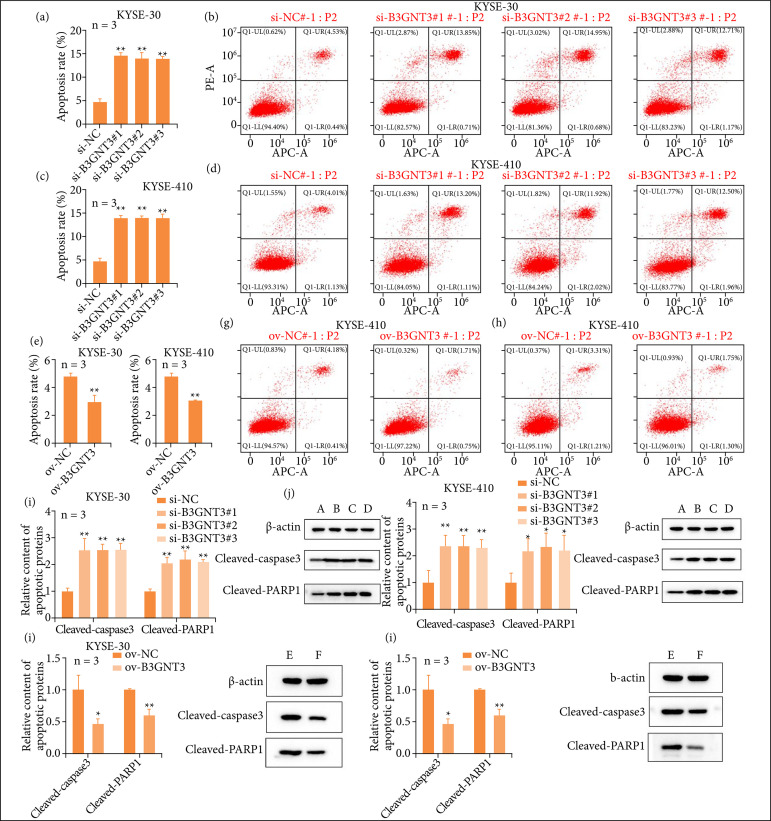
B3GNT3 inhibitor ESCA cells apoptosis. **(a–d)** The apoptosis rates of KYSE-30 and KYSE-410 cells. (**e-h**)The apoptosis rates of overexpression the B3GNT3 in KYSE-30 and KYSE-410 cells. **(i, j)** Relative expression of apoptotic proteins in KYSE-30 and KYSE-410 cells after manipulating B3GNT3 expression. **(k, l)** Relative expression of apoptotic proteins in KYSE-30 and KYSE-410 cells after overexpression B3GNT3 expression. Compared with si-NC, **p < 0.01, * p < 0.05. Compared with ov-NC, **p < 0.01, *p < 0.05. A: si-NC, B: si-B3GNT3

Flow cytometry results showed that the apoptosis rate of KYSE-30 and KYSE-410 cells increased significantly after manipulating B3GNT3 expression (Fig. 4a–d), while overexpression the B3GNT significantly reduced apoptosis rate (Fig. 4e–h). The levels of cleaved-caspase3 and Cleaved-PARP1 increased in KYSE-30 and KYSE-410 cells after manipulating B3GNT3 expression (Fig. 4i, j), while overexpression the B3GNT significantly reduced (Fig. 4k, l). These results suggested that B3GNT3 repaired ESCC cells damage.

### B3GNT3 promoted the growth of ESCA cell in vivo

**Figure 5:**
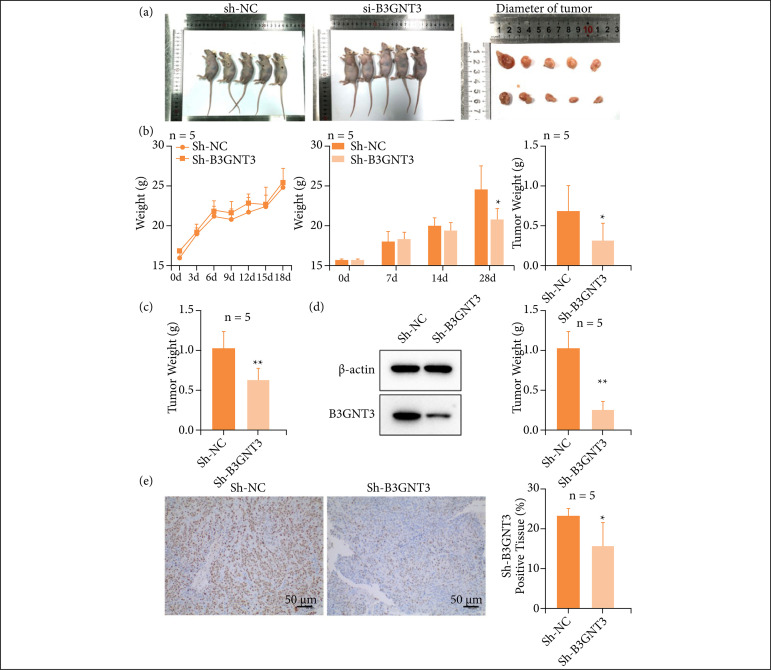
The ability of B3GNT3 to promote the proliferation of ESCC cells in vivo. **(a)** Comparison of tumor volume in nude mice. Body weight curve of nude mice; Tumor growth histogram; **(b)** Tumor weight histogram of nude mice **(B)**. **(c, d)** PCR and WB was used to determine the mRNA expression of Sh-B3GNT3 (C, D). **(e)** The tumor marker Ki-67 was detected by immunohistochemistry. **p < 0.01, **p < 0.01 vs. Sh-NC.

The tumor-bearing assay in nude mice was used to detect whether B3GNT3 promoted the growth of ESCC in vivo. When KYSE-30 cells were injected into the armpit of the right forelimb of nude mice, macroscopic tumor clumps appeared on day 7 after injection, and tumor volumes were measured with vernier calipers every 7 days thereafter. After 4 weeks of continuous weighing, the tumor was removed, weighed, and photographed. Results As shown in Fig. 5, the tumor volume in Sh-B3GNT3 group was significantly smaller than that in Sh-NC group (Fig. 5a), and the weight change curve showed that Sh-NC group was lower than Sh-B3GNT3 group. Tumor growth showed that the tumor volume of Sh-NC mice increased rapidly, while Sh-B3GNT3 mice grew slowly (p < 0.05). The tumor weight of nude mice in Sh-NC group was significantly higher than that of Sh-B3GNT3 group (p < 0.05) (Fig. 5b). qRT-PCR and WB results showed that B3GNT3 was highly expressed in Sh-NC group (p < 0.01) (Fig. 5c, d). Immunohistochemistry showed that the tumor marker Ki-67 was highly expressed in the Sh-NC group (p < 0.01) (Fig. 5e). These results indicate that B3GNT3 can promote the growth of ESCC cells in vivo.

### Functional enrichment analysis

**Figure 6:**
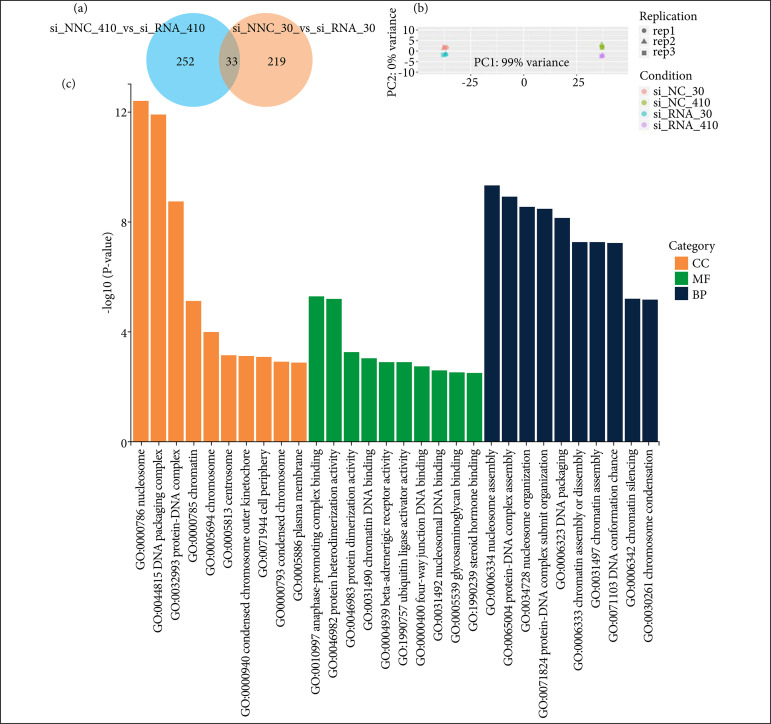
Transcriptome sequencing of B3GNT3 in ESCC cell lines. **(a)** Coorder Venn diagram between samples; **(b)** principal component analysis (PCA); **(c)** Gene Ontology gene function enrichment analysis. Rich factor represents the proportion of enriched differential genes in the background genes of the pathway, and the abscissa is the name of the pathway with higher enrichment degree.

To better understand the molecular mechanism by which B3GNT3 promotes the proliferation, migration and invasion of ESCC cells, RNA transcriptome sequencing (RNA-Seq) was performed to detect differentially expressed genes in B3GNT3-treated cells and control cells. The detailed analysis of transcriptome sequencing results showed that 504 genes were detected in B3GNT3 and control group cells (Fig. 6a). Principal component analysis showed that the clustering between samples was good, and 285 differentially expressed genes were identified in the si_NC_410 (si_NC_ KYSE-410) group, of which 128 were up-regulated and 157 were down-regulated. A total of 252 differentially expressed genes were identified in the si_NC_30 (si_NC_ KYSE-30) group, of which 86 were up-regulated and 166 were down-regulated (Fig. 6b). In addition, through GO gene function enrichment analysis (Fig. 6c) of candidate target genes, the potential functions corresponding to the expression profile of candidate target genes were grasped as a whole. The candidate target genes were found to be concentrated in nucleosome, DNA packaging complex, nucleosome assembly and other cellular functions, and prepare for subsequent experiments.

**Figure 7:**
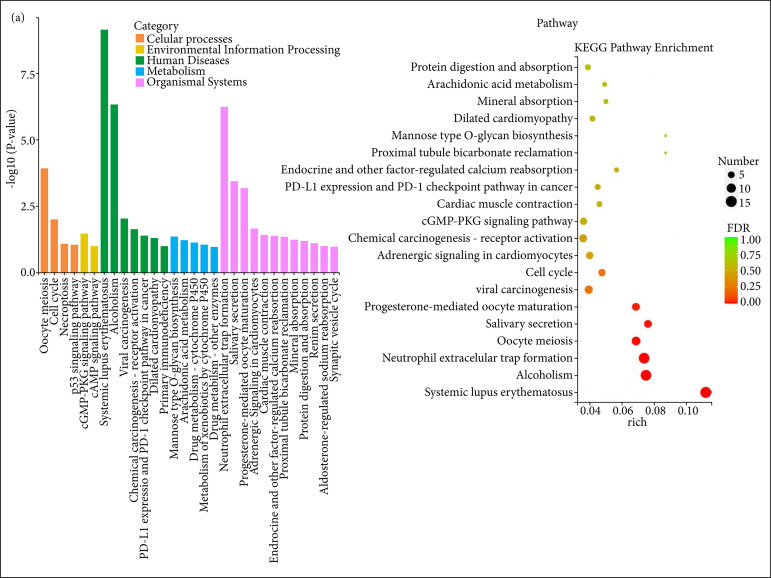
KEGG pathway enrichment analysis of B3GNT3 in ESCC cell lines. **(a)** KEGG enrichment analysis; **(b)** KEGG pathway enrichment bubble diagram.

In order to further explore the mechanism by which B3GNT3 regulates the downstream target genes of mRNAs to inhibit the growth of ESCC, the KEGG pathway mainly involved in the candidate target genes was divided into five branches: cell process, Environmental Information Processing, Human Disease, Metabolism, Organismal Systems (Fig. 7a). Further KEGG Pathway enrichment bubble diagram (Fig. 7b) showed that the differential genes were enriched in a variety of cancer signaling pathways, including Viral carcinogenesis, Neutrophil extracellular traps, Systemic lupus erythematosus, etc. Combined with KEGG Pathway classification analysis and enrichment analysis, B3GNT3 may be involved in a variety of carcinogenesis pathways in ESCC cells by regulating the target genes of mRNAs.

In Fig. 7, the abscissa Rich factor represents the proportion of enriched differential genes in the background genes of the pathway, and the abscissa is the name of the pathway with higher enrichment degree; the size of the point in Fig. 7 indicates the number of enriched differential genes, the larger the volume of the point indicates the more enriched genes, the color indicates the p value, and the smaller the p value indicates the more obvious enrichment of the pathway.

## Discussion

The relationship between B3GNT3 and tumorigenesis has been confirmed to a certain extent, but the role of B3GNT3 in the development of ESCA is not very clear. To the best of our knowledge, this study is the first to comprehensively analyze the correlation between B3GNT3 expression and clinicopathological features and prognosis of ESCA. In this study, B3GNT3 expression was significantly upregulated in cancer tissues in association with ESCA adjacent tissues or normal esophageal tissues, which is consistent with previous findings. At the same time, patients with high expression of B3GNT3 have significantly shorter survival time and poorer tissue differentiation, which suggests that B3GNT3 can be used as a reference index for the prognosis of ESCA. Based on these findings, we further explored the role of B3GNT3 in the progression of ESCA.

Previous studies have shown that B3GNT3 levels are positively correlated with lymph node metastasis in non-small cell lung cancer and cervical cancer, suggesting that B3GNT3 may play a role in the progression of these two tumors^14^^,^ 15 In addition, Ho et al. showed that overexpression of B3GNT3 could inhibit the invasion and migration of neuroblastoma cells^24^. In this study, the effects of B3GNT3 on the proliferation, migration, invasion and apoptosis of ESCC cells were detected by CCK-8, transwell, flow cytometry, WB and qPCR. The results showed that the proliferation ability, migration and invasion ability of KYSE-30 and KYSE-410 cells with B3GNT3 interference were significantly lower than those of the control group, and the overexpression of B3GNT3 in KYSE-30 and KYSE-410 cells had the opposite effect. After silencing B3GNT3 expression in ESCC cell lines, the growth of both cell lines was inhibited and the invasiveness was decreased. Overexpression of B3GNT3 increased cell growth and invasiveness in both cell lines. This study demonstrated that genetic alterations in B3GNT3 genes contribute to ESCA progression. The outcomes suggest that B3GNT3 might be a candidate marker for the diagnosis and treatment of patients with ESCA. In vitro experiments further verified that tumor marker Ki-67 was significantly reduced after effectively knocking down the expression level of B3GNT3 in ESCA cells with sh-RNA. These findings suggest that B3GNT3 plays a potential role in tumor immune infiltration in ESCA. In this study, mRNA-seq revealed that the immunosuppressive state in the tumor microenvironment of ESCC deteriorated with tumor progression, suggesting that B3GNT3 plays a potential role in the immunoreaction of ESCA.

With the development of high-throughput sequencing technology, the mechanism of epigenetics in the occurrence and development of ESCA has been confirmed^25^^–^ 27. B3GNT3 may be involved in viral carcinogenesis, neutrophil extracellular traps and systemic lupus erythematosus signaling pathways of ESCA by transcriptome sequencing. Indicating that the immunosuppressive state in the ESCC tumor microenvironment deteriorates with tumor progression. This study will help to understand the tumor cell specificity of patients with ESCA.

## Conclusion

B3GNT3 acts as an oncogene by participating in the growth, invasion and migration of ESCA cells. This study will help to understand the tumor microenvironment and cellular heterogeneity of ESCA patients, and provide valuable data for further exploring the pathogenesis of ESCA and identifying potential therapeutic targets in the future.

## Data Availability

The data will be available upon request.
